# The Subcellular Dynamics of the Gs-Linked Receptor GPR3 Contribute to the Local Activation of PKA in Cerebellar Granular Neurons

**DOI:** 10.1371/journal.pone.0147466

**Published:** 2016-01-22

**Authors:** Tatsuhiro Miyagi, Shigeru Tanaka, Izumi Hide, Toshihiko Shirafuji, Norio Sakai

**Affiliations:** Department of Molecular and Pharmacological Neuroscience, Institute of Biomedical & Health Sciences, Hiroshima University, Hiroshima 734–8551, Japan; Indiana University School of Medicine, UNITED STATES

## Abstract

G-protein-coupled receptor (GPR) 3 is a member of the GPR family that constitutively activates adenylate cyclase. We have reported that the expression of GPR3 in cerebellar granular neurons (CGNs) contributes to neurite outgrowth and modulates neuronal proliferation and survival. To further identify its role, we have analyzed the precise distribution and local functions of GPR3 in neurons. The fluorescently tagged GPR3 protein was distributed in the plasma membrane, the Golgi body, and the endosomes. In addition, we have revealed that the plasma membrane expression of GPR3 functionally up-regulated the levels of PKA, as measured by a PKA FRET indicator. Next, we asked if the PKA activity was modulated by the expression of GPR3 in CGNs. PKA activity was highly modulated at the neurite tips compared to the soma. In addition, the PKA activity at the neurite tips was up-regulated when GPR3 was transfected into the cells. However, local PKA activity was decreased when endogenous GPR3 was suppressed by a GPR3 siRNA. Finally, we determined the local dynamics of GPR3 in CGNs using time-lapse analysis. Surprisingly, the fluorescent GPR3 puncta were transported along the neurite in both directions over time. In addition, the anterograde movements of the GPR3 puncta in the neurite were significantly inhibited by actin or microtubule polymerization inhibitors and were also disturbed by the Myosin II inhibitor blebbistatin. Moreover, the PKA activity at the tips of the neurites was decreased when blebbistatin was administered. These results suggested that GPR3 was transported along the neurite and contributed to the local activation of PKA in CGN development. The local dynamics of GPR3 in CGNs may affect local neuronal functions, including neuronal differentiation and maturation.

## Introduction

G-protein coupled receptors (GPRs) are the most abundant membrane proteins and have been reported to form a large family. Among them, GPR3, GPR6, and GPR12 constitutively activate the Gs protein, which resulted in constitutively increased intracellular cAMP [1]. These receptors are largely expressed in the brain, with the exception of GPR3, which shows additional expression in oocytes and testis. GPR12 is also expressed in the liver [2,3]. We have reported that GPR3 expression in cerebellar granular neurons (CGNs) is associated with neurite outgrowth, the modulation of premature neuronal proliferation, and neuronal survival [3–5]. Furthermore, recent reports implicated the involvement of GPR3 in amyloid-beta production [6,7], emotional-like responses [8], neuropathic pain [9], and cocaine reinforcement [10]. However, the physiological function of GPR3 has not been fully elucidated.

In the rodent brain, in situ hybridization showed that GPR3 is expressed in the medial habenular nucleus, cerebral cortex, hippocampus, olfactory bulb, and striatum [2]. Later, GPR3 is also expressed in the internal layer of the cerebellum. Moreover, the expression of GPR3 is increasingly expressed in CGNs following development in vitro and in vivo [5]. At the single cell level, GPR3 is expressed in the plasma membrane, as demonstrated by fluorescently tagged GPR3 transfection in oocytes and HEK293 cells [3,11]. Recently, GPR3 was shown to be internalized by a G-protein-coupled receptor kinase (GRK)2- and arrestin2-dependent mechanism in HEK293 cells [12]. However, the precise distributions and local functions of GPR3 in neurons have not been fully understood.

Neurons possess a highly polarized structure consisting of an axon and dendrites. To maintain neuronal homeostasis and activity, various protein complexes, mitochondria, mRNAs are transported to the axon and dendrites [13]. In contrast to non-polarized cells, neurons have a long axon; in particular, the motor neuron axon extends over one meter to the neurite tips in humans. Therefore, it is possible that the transportation of various cargos in neurites is important for neuronal homeostasis, function, and survival. Myosin, kinesin and the dynein superfamily have been reported to play a role in the selective transport of cargo proteins in neurons [13]. Kinesin and dynein move along the microtubules, whereas the myosin super family of motor proteins transport cargo along actin filaments. In kinesin-mediated transport, the N-terminal motor domain KIFs transport cargo toward the plus ends, whereas the C-terminal motor domain KIFs and dynein transport cargo toward minus ends.

In neurons, kinesin family proteins are involved in the transportation of various organelles and proteins, such as synaptic proteins [14], TrkB [15], AMPA receptor [16], GABA(A) receptor [17], mitochondria [18] and the amyloid precursor protein [19]. In developing neurons, kinesin-1 family proteins are concentrated at the neurite tips [20], and transport cargos, such as TrkB, via the CRMP2-Slc1 complex [15]. On the other hand, myosin super family motor proteins also play a role in neuronal transport in the synaptic regions [21,22] and in neuronal migration [23,24]. More recently, Myosin II has been shown to drive cortical F-actin flow, which provides the net forward transport of proteins in the plasma membrane [25]. However, GPR3 transport in neuronal cells has been poorly understood.

In the present study, we analyzed the distribution of GPR3 in rodent mouse brain. We further analyzed the distribution of GPR3 in CGNs by transfecting fluorescently tagged GPR3 expression vectors. Time-lapse analysis of these cells revealed that the GPR3 florescent puncta moved along the neurite. The local dynamics of GPR3 were significantly correlated with local PKA activation at the neurite tips in CGNs. Our current studies suggested that GPR3 may contribute to the local activation of PKA in CGN development.

## Materials and Methods

### Animals

C57BL/6 mice and Wistar rats were obtained from SLC (Shizuoka, Japan). GPR3-knockout mice (GPR3-/-; LacZ+/+ mice) were generated from cryopreserved embryos, which were obtained from MMRRC (Bar Harbor, ME) and resuscitated at the Institute of Laboratory Animal Science, Hiroshima University under the license of Deltagen, as previously described (San Mateo, CA) [4]. All experiments were approved by the Animal Care and Use Committee of Hiroshima University (approval number A13-155).

### Materials

Dulbecco’s modified Eagle medium (DMEM), Ham's F12 Medium, Neurobasal A medium, B27 supplement, GlutaMAX, fetal bovine serum, Alexa Fluor secondary antibodies, LysoTracker, MitoTracker, Alexa Fluor 633-conjugated WGA, CellMask, and Hoechst33342 were obtained from Life Technologies (Carlsbad, CA). The penicillin/streptomycin solution and polyethyleneimine were from Nakalai Tesque (Kyoto, Japan). The HaloTag vectors and HaloTag ligands were from Promega (Madison, WI). The anti-KDEL antibody was from Enzo Life Sciences (Farmingdale, NY). The anti-Rab5 and anti-Rab7 antibodies were from Cell Signaling Technology (Danvers, MA). The G Silencer siRNA against rat GPR3 (5’-CCUACUACUGAGAGACAACtt-3’/5’-GUUGUGUCUGAGUAGUAGGtg-3’) and the negative control #1 siRNA were purchased from Ambion, Inc. (Austin, TX). The glass-bottomed culture dishes (35-mm diameter) were from MatTek (Ashland, MA) and the 8-well chamber slides were from AGC Techno Glass (Shizuoka, Japan). All other reagents or chemicals were from Sigma-Aldrich (St. Louis, MO), unless otherwise indicated.

### Plasmid Constructs

The full-length mouse GPR3 cDNA was amplified by PCR and inserted in frame into the HaloTag expression vector, which was designated as pGPR3-HT. In the same way, a PCR fragment of mouse GPR3 was inserted in frame with monomeric GFP into a FLAG-tagged expression vector, which was designated as pGPR3-mAGFL. pYFP-mem was from Life Technologies. The expression vector for the FRET-based PKA sensor AKAR3-EV [26] was kindly provided Michiyuki Matsuda (Kyoto University).

### X-gal staining

After anaesthetizing the GPR3 heterozygote knockout mice, the animals were transcardially perfused with an ice-cold 4% paraformaldehyde-PBS solution. The brains were then removed and post-fixed in 4% paraformaldehyde-PBS for eight hours. Coronal or sagittal sections were cut using a vibratome (Dosaka EM, Kyoto, Japan). The sections were then immersed in 0.05% glutaraldehyde (EM grade) for 10 min for additional fixation, followed by two washes with phosphate-buffered saline. The sections were immersed in an X-gal staining solution (1 mM MgCl_2_, 3 mM potassium ferricyanide, 3 mM potassium ferrocyanide, and 1 mg/ml X-gal in phosphate buffer) for one hour at 37°C. The X-gal reaction was terminated by washing in PBS(-) followed by 4% paraformaldehyde. The images were captured on a BZ-9000 microscope (Keyence, Tokyo, Japan) using the tiling scan mode, and then reconstructed to a single image with built-in image joining software.

### Cell culture, CGN isolation, and transfection

The SH-SY5Y human neuroblastoma cell line was obtained from ATCC (American Tissue Culture Collection, Manassas, VA). SH-SY5Y cells were grown in DMEM/F-12 (1:1) media supplemented with 10% fetal bovine serum and 1% penicillin-streptomycin. The CGNs were isolated from Wistar rats or C57BL/6 mice, as detailed in elsewhere [3]. Briefly, the entire cerebellum was removed from postnatal day 7 (P7) pups and dissociated using the Worthington Papain Dissociation System (Worthington Biochemical, Lakewood, NJ). The dissociated cells were then separated using two-step Percoll gradient (35%/ 60%) centrifugation. After centrifugation at 2,000 ×*g* for 10 min, the granule neuron-enriched fraction was collected from between the 35% and 60% Percoll layers. The isolated neurons were washed once with PBS and suspended in DMEM medium supplemented with 10% fetal bovine serum and plated at 5x10^5^/cm^2^ onto 0.03% polyethyleneimine-coated glass bottom dishes and 8-well chamber slides. Four hours after cell plating, the culture media was replaced with CGN culture medium: Neurobasal A media supplemented with 2% B27 supplement, 200 μM of GlutaMAX and 1% penicillin-streptomycin. Using this protocol, 95–99% of the cultured cells were neurons. All cells were cultured in a humidified atmosphere containing 5% CO_2_ at 37°C.

For the imaging experiments, the fluorescently tagged GPR3 expression vectors were transfected into the CGNs or cell lines using a NEPA21 Super Electroporator (Napa Gene, Chiba, Japan) according to the manufacturer’s protocols. Briefly, 5x10^6^ cells were counted using a TC20 automated cell counter (BioRad, Hercules, CA) and re-suspended in 100 μl of serum free Neurobasal A medium, mixed with 50 μg of plasmid DNA or 1.5 μg of siRNA, and transferred into a cuvette (2 mm GAP) (Napa Gene). The electroporation for the CGNs and SH-SY5Y cells was performed using the following conditions: for the poring pulse—175 V, 2.5 msec pulse length, 50 msec pulse interval, 4 pulses, and a 10% decay rate; and for the transfer pulse—20 V, 50 msec pulse length, 50 msec pulse interval, 5 pulses, and a 40% decay rate. After electroporation, the cells were immediately rescued with 600 μl of DMEM medium containing 10% FBS and plated onto the glass bottomed dishes at a density of 5x10^5^ cells/ well. Four hours after plating, the culture medium was replaced with fresh CGN culture medium. Using this protocol, at least 80% of cells were viable on the following day (data not shown).

### Analyses of GPR3 localization and time-lapse imaging

To analyze the localization of GPR3, the CGNs were transfected with pGPR3-HT or pGPR3-mAGFL plasmids as described above. Forty-eight hours after transfection, the cells were fixed with 4% paraformaldehyde. To determine the co-localization of GPR3 with the endoplasmic reticulum and endosomes, GPR3-transfected CGNs were immunostained with anti-KDEL (1:100), anti-Rab5 (1:100), and anti-Rab7 antibodies (1:100), respectively, followed by Alexa Fluor-conjugated goat anti-mouse and anti-rabbit IgG antibodies (1:400), respectively. To analyze the localization of GPR3 in the Golgi bodies, pGPR3-mAGFL-transfected CGNs were also labeled with WGA633, a Golgi body marker, for 30 min before fixation. To analyze the plasma membrane localization of GPR3, the CGNs were co-transfected with pGPR3-HT and pYFP-mem, a marker for the plasma membrane. To further confirm the plasma membrane localization of GPR3, pGPR3-mAGFL-transfected CGNs were also labeled with CellMask, another plasma membrane marker, for 30 min before fixation. The labeled cells were counterstained with Hoechst 33342. The co-localization of the fluorescently tagged GPR3 with the organelle markers was evaluated using a Zeiss LSM510 META confocal microscope (Carl Zeiss, Oberkochen, Germany). The fluorescent intensity was analyzed by “line-profiling” using MetaMorph software. Briefly, to detect the PKA activities in the plasma membrane and cellular organelles, a line was drawn across the cell body that avoided the nucleus, and then a list of intensity values was obtained. To detect the PKA activities in the neurites or neurite tips, the line was drawn across the neurites or growth cones at the neurite tips, respectively.

To capture the time-lapse images, the neurons were transfected with pGPR3-HT or pGPR3-mAGFL plasmids. Forty-eight hours after transfection, the cells were imaged with a BZ-9000 fluorescent microscope (Keyence, Tokyo, Japan). The microscope was equipped with a CO_2_ incubation chamber (Tokai Hit, Shizuoka, Japan), and the cultured cells were maintained in a humidified atmosphere containing 5% CO_2_ at 37°C while the images were captured.

### PKA FRET analysis

For the PKA FRET analysis, the neurons were co-transfected with AKAR3-EV and a GPR3 expression plasmid. Forty-eight hours after transfection, the cells were imaged with a BZ-9000 fluorescent microscope equipped with an oil immersion 60x Nikon Plan Apo VC objective lens with a numerical aperture of 1.4 using 2 x 2 binning on built-in cooled CCD camera with a 2.5x digital zoom. While capturing the images, the culture dishes were kept in a humidified atmosphere containing 5% CO_2_ at 37°C. CFP and FRET images were obtained with two sets of filter cubes; one with the 438/24 nm excitation filter, 458nm dichroic mirror, and the 483/32nm emission filter for CFP channel, and the second one with the 438/24 nm excitation filter, 458nm dichroic mirror, and the 542/27nm emission filter for FRET channel (Semrock, Lake Forest, IL). Samples were exposed 83ms for both CFP and FRET with a 20% transmittance with a natural density filter in the excitation light passed through diffusion filter from the 120W Hg arc lamp source. To obtain the FRET level of PKA in the fully activated condition, the FRET/CFP images were also captured after administering dbcAMP (final conc. 1 mM) into the culture medium with a bath application. After each measurement, FRET and CFP fluorescent values were corrected for bleedthrough (S2B and S2C Fig) and background as described [27,28]. The corrected FRET/CFP ratio images were analyzed by the MetaMorph software (Universal Imaging, West Chester, PA).

To analyze the distribution of PKA activity in a single cell, the CGNs were transfected with AKAR3-EV/pGPR3-HT or AKAR3-EV/mock vectors. Forty-eight hours after transfection, the CGNs were stained with CellMask (plasma membrane), ER tracker (ER), WGA633 (Golgi body), LysoTracker (lysosome), and MitoTracker (mitochondria), respectively. The FRET/CFP ratios in the images were visualized and analyzed by the MetaMorph software.

### Data analysis

The data are expressed as the means ± SEM. The statistical analyses were performed using one-way ANOVA followed by Fisher's PLSD test, unless otherwise indicated. A value of p < 0.05 was considered significant.

## Results

### The distributions and subcellular localizations of GPR3 in mouse brain

In situ hybridization analysis reported that GPR3 is distributed in the medial habenular nucleus, cerebral cortex, hippocampus, olfactory bulb, striatum, and cerebellum in the mouse brain [2]. However, the precise distribution of GPR3 in the rodent CNS has not been fully elucidated. To clarify the precise distribution of GPR3 in the central nervous system, we employed GPR3-/-; LacZ+/+ mice, where the GPR3 gene locus is genetically substituted by the β-galactosidase gene, to evaluate the distribution of GPR3 promoter activity. X-gal staining revealed that the activity of the GPR3 promoter increased in the medial habenular nucleus, the CA2 region of hippocampus, the thalamus and the pontine nucleus, but was rather weak in the striatum, cortex, cerebellum, medulla oblongata, brain stem, and spinal cord (S1 Fig). High magnification images of the cortex, hippocampus and cerebellum revealed that most of the X-gal-stained cells were neurons, based on their morphology and distribution. These results suggested that GPR3 is primarily distributed in the neurons of various regions of the central nervous system.

**Fig 1:**
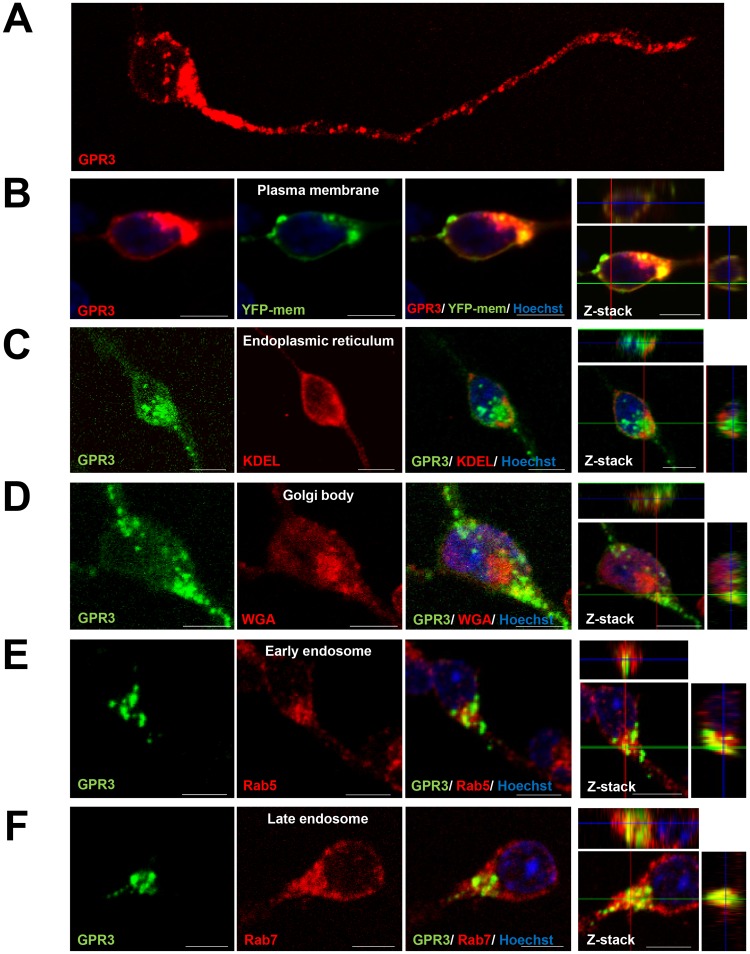
The subcellular distribution of fluorescently tagged GPR3 in CGNs. (A) We employed a GPR3-HT expression vector to analyze the localization of GPR3. After transfecting the GPR3-HT vector into the CGNs, the cells were labeled with the TMR HaloTag ligand at forty-eight hours after transfection. A representative image was shown. (B) To evaluate the localization of GPR3 at the plasma membrane, the CGNs were co-transfected with the GPR3-HT and YFP-mem expression vectors, followed by TMR HaloTag ligand labeling. YFP-mem fluorescence was shown in the green pseudo-color to easily visualize the co-localization. (C-F) To evaluate the localization of GPR3 in the endoplasmic reticulum, Golgi body, and endosomes, the GPR3-mAGFL expression vector was transfected into the CGNs. Forty-eight hours after transfection, the cells were immunostained with various organelle markers: anti-KDEL antibody (endoplasmic reticulum), WGA633 (Golgi body), anti-Rab5 antibody (early endosome), and anti-Rab7 antibody (late endosome). Co-localization of GPR3 with the organelle markers was evaluated by confocal microscopy using built-in Z-stack analysis software. Scale bar = 10 μm.

Next, we examined the subcellular localization of GPR3 in neuronal cells. To address this, fluorescently tagged GPR3 expression plasmids were transfected in CGNs, and we evaluated the co-localization of the fluorescently tagged GPR3 with organelle markers. The fluorescent GPR3 fusion protein was observed along the plasma membrane and in the cytosol (Fig 1A). The plasma membrane expression of the GPR3 fusion protein was confirmed by co-localization with YFP-mem, which is a marker for the plasma membrane (Fig 1B). Moreover, the GPR3 fusion protein was co-localized with the markers for the Golgi body and endosomes, but was not co-localized with the marker for the endoplasmic reticulum (Fig 1C–1F). Fluorescent GPR3 puncta were also observed in neurites; however, these puncta were not co-localized with the markers for early and late endosomes (data not shown). Similar subcellular distributions of GPR3 were observed when the fluorescently tagged GPR3 expression vector was transfected in SH-SY5Y cells (data not shown).

### Subcellular analyses of GPR3-mediated PKA activity in CGNs

GPR3 has been thought to be a constitutive activator of intracellular cAMP in the absence of ligand in neuronal [3] and non-neuronal cells [1,29]. In addition, a recent report indicated that the plasma membrane expression of GPR3 functionally up-regulates the levels of intracellular cAMP in HEK293 cells [12]. However, it remains unclear whether the activation of PKA is modulated by GPR3, and where PKA is activated by GPR3 in a single neuron. To address this issue, we introduced the FRET-based PKA indicator AKAR3-EV [26] to evaluate the PKA activity in a single cell.

**Fig 2:**
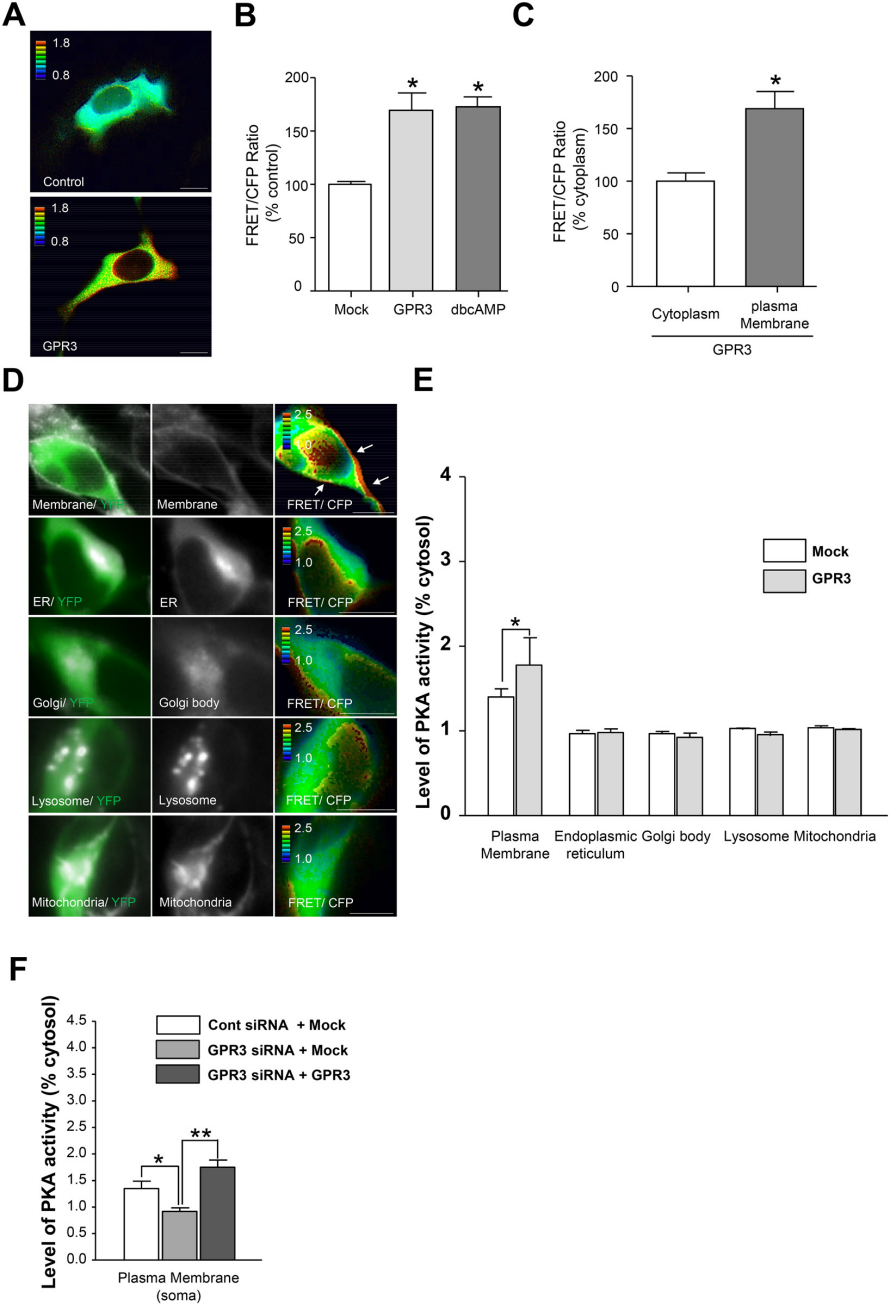
The GPR3-mediated modulation of PKA activity was analyzed with a PKA FRET indicator. (A-C) HEK293 cells were co-transfected with the AKAR3-EV and GPR3 expression plasmids. AKAR3-EV and the mock vector were co-transfected as a control. Forty-eight hours after transfection, the CFP and FRET images were captured using a fluorescent microscope. Some of the AKAR3-EV/mock vector co-transfected cells were treated with 1 mM dbcAMP 15 min before the images were captured. The FRET/CFP ratios in the images were analyzed by the MetaMorph software. (A) Representative images of cells transfected with either the mock vector (top) or the GPR3 vector (bottom) are shown. (B) The YFP/CFP ratio in each group were analyzed identically in each dish (n = 8). The values are expressed as the percentage of the FRET/CFP ratio in the GPR3-transfected or dbcAMP-treated cells compared to the mock-transfected cells, i.e., the percentage of the control vector-transfected cells. The means ± SEM were calculated for each condition. The asterisk (*) represents p < 0.001 compared to the cells transfected with the mock vector. (C) The FRET/CFP ratio in the cytoplasm and plasma membrane were evaluated in a single GPR3-transfected cell. The asterisk (*) represents p < 0.001 compared to the values for the cytoplasm. (D-E) The PKA activity in GPR3-expressing CGNs was evaluated by AKAR3-EV. CGNs were co-transfected with mock/AKAR3-EV or pGPR3-HT/AKAR3-EV expression plasmids. Forty-eight hours after transfection, the CGNs were stained with organelle-specific markers for the plasma membrane, ER, Golgi body, lysosome, and mitochondria. (D) Representative images of CGNs stained with CellMask (plasma membrane), ER tracker (ER), WGA633 (Golgi body), LysoTracker (lysosome), and MitoTracker (mitochondria) were shown (middle raw). The CFP/YFP ratios in the images were visualized and analyzed by the MetaMorph software (right raw). (E) The FRET/CFP ratios were compared in each organelle of the mock/AKAR3-EV- or pGPR3-HT/AKAR3-EV-transfected CGNs. The FRET/CFP ratios were significantly increased in the plasma membrane of the GPR3-HT-expressing CGNs compared to the mock-transfected CGNs. The data represent the means ± SEM for each condition (n = 6). The asterisk (*) represents p < 0.05. (F) The PKA activity in the plasma membrane around soma was analyzed in CGNs transfected with control siRNA+Mock, GPR3 siRNA+Mock, and GPR3 siRNA+ pGPR3-HT, respectively. Forty-eight hours after transfection, the FRET/CFP images were taken. The FRET/CFP ratio in each group were analyzed identically. The data represent the means ± SEM for each condition (n = 10). The asterisk (*) represents p < 0.05 and the double asterisk (**) represents p < 0.005.

First, we confirmed the GPR3-mediated PKA activation using AKAR3-EV in HEK293 cells. HEK293 cells were co-transfected with a GPR3 expression vector and AKAR3-EV, and the FRET/CFP ratio was evaluated 48 hours after transfection. The FRET/CFP ratio in the cytosol was significantly increased in the GPR3-expressing HEK293 cells compared to the mock vector-transfected HEK293 cells (Fig 2A and 2B). The elevation of the FRET/CFP ratio by GPR3 was similar to the cells that were treated with 1 mM dbcAMP. Furthermore, the FRET/CFP ratios were relatively higher in the plasma membrane compared to the cytoplasm in the GPR3-expressing HEK293 cells (Fig 2C). We further confirmed whether the FRET phenomena of AKAR3-EV has really occurred in our assay system using acceptor photo bleaching method. Photo bleaching the acceptor fluorophore affects on dequenching the donor fluorescence, thereby proving that FRET phenomena has occurred retrospectively [30]. FRET fluorescent was decreased after acceptor was bleached, whereas CFP fluorescent intensity was increased after acceptor photo bleaching (S2A Fig). We thus concluded that the GPR3-mediated PKA activation could be detected by the AKAR3-EV FRET sensor in a single cell. We then applied these systems to the CGNs to evaluate the PKA activity in a single neuron. The CGNs were co-transfected with a GPR3 expression vector and AKAR3-EV, and the FRET/CFP ratio was evaluated 48 hours after transfection. PKA activity was significantly increased along the plasma membrane in the GPR3-expressing cells compared to the mock vector-transfected cells (Fig 2D and 2E). However, PKA activation was not detected in intracellular organelles, such as the endoplasmic reticulum, Golgi body, lysosome, and mitochondria. Then, we examined whether inhibiting endogenous GPR3 expression affected the GPR3-mediated PKA activity in the plasma membrane. Our previous reports demonstrated that endogenous GPR3 expression was reduced to 60% of the control mRNA levels by the GPR3 siRNA [3]. PKA activity was significantly decreased in the plasma membrane around soma in the GPR3 siRNA-transfected CGNs compared to the control siRNA-transfected CGNs (Fig 2F). In addition, the GPR3 siRNA-mediated reduction of the PKA activity in the plasma membrane was rescued when a siRNA-resistant GPR3 expression vector was co-transfected with the GPR3 siRNA (Fig 2F). These results suggested that the expression of GPR3 in the plasma membrane contributes to the functional activation of PKA in adjacent areas in CGNs.

### The PKA activity was highly modulated at the tips of neurite by GPR3 expression in CGNs

**Fig 3:**
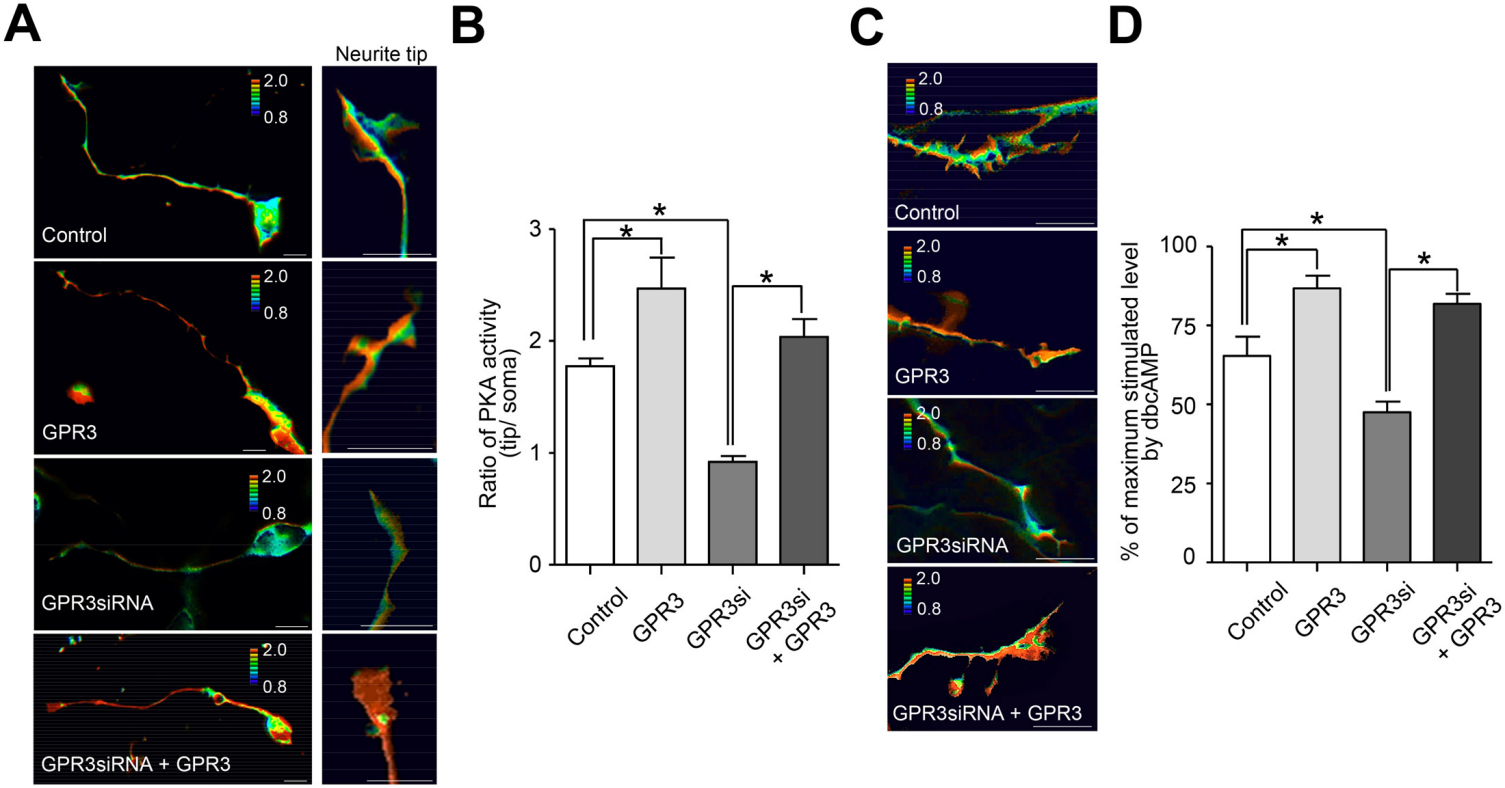
The local PKA activity was modulated by the expression of GPR3 in the CGNs. (A-B) The local PKA activity was compared between the soma and neurite tips in GPR3-transfected CGNs. The CGNs were transfected with Mock+control siRNA, pGPR3-HT+control siRNA, Mock+GPR3 siRNA, and pGPR3-HT+GPR3 siRNA, respectively. Forty-eight hours after transfection, the FRET/CFP images were captured using a fluorescent microscope. After capturing the images, the FRET/CFP intensity ratio was calculated and compared between the soma and neurite tips. (A) Representative FRET/CFP images from CGNs in each condition were shown (left raw). Magnifications of neurite tip were also shown (right raw) (B) The ratios of the PKA activity (tip to soma) in each condition were analyzed identically. The data represent the means ± SEM for each condition (n = 7). The asterisk (*) represents p < 0.005. (C-D) The PKA activity at the neurite tips was analyzed in CGNs transfected with Mock+control siRNA, pGPR3-HT+control siRNA, Mock+GPR3 siRNA, and pGPR3-HT+GPR3 siRNA, respectively. Forty-eight hours after transfection, the FRET/CFP images were captured using a fluorescent microscope. After capturing the images, the cells were treated with 1 mM dbcAMP for 15 min, and FRET/CFP images were captured again in the same cell to evaluated fully activated PKA. (C) Representative FRET/CFP images from the neurite tips of CGNs in each condition were shown. (D) The FRET/CFP ratio in each group were analyzed identically. The FRET/CFP ratios are expressed as the percentage of the maximum level stimulated by 1 mM dbcAMP in each cell. The data represent the means ± SEM for each condition (n = 9). The asterisk (*) represents p < 0.005.

Neurons possess highly polarized structures during neuronal maturation. Our previous reports indicated that GPR3 expression is up-regulated in CGNs during neuronal development [4,5]. Next, we asked whether the PKA activities in the plasma membrane differ between the soma and the neurite tips. When the CGNs were transfected with the mock vector, the PKA activity in the plasma membrane was increased at the neurite tips compared to the soma (Fig 3A and 3B). In addition, the PKA activity at the neurite tips was further up-regulated when the CGNs were transfected with the GPR3 expression vector. Then, we examined whether inhibiting endogenous GPR3 expression affected the local GPR3-mediated PKA activity. The increased PKA activity at the neurite tips was significantly reduced in the GPR3 siRNA-transfected CGNs compared to the control siRNA-transfected CGNs (Fig 3A and 3B). In addition, the GPR3 siRNA-mediated reduction of the PKA activity at the neurite tips was rescued when a siRNA-resistant GPR3 expression vector was co-transfected with the GPR3 siRNA.

We further evaluated whether the local PKA activity at the neurite tips was modulated by the expression of GPR3. CGNs were co-transfected with a GPR3 expression vector and AKAR3-EV, and FRET/CFP images were taken 48 hours after transfection. After the images were captured, the cells were treated with 1 mM dbcAMP for 15 min, and another set of FRET/CFP images were captured in the same cell to evaluate the fully activated PKA. The FRET/CFP ratios are expressed as the percentage of the maximum level stimulated by 1 mM dbcAMP in each cell. The FRET/CFP ratio in the plasma membranes of the neurite tips was significantly increased in GPR3-transfected CGNs compared to the mock vector-transfected CGNs (Fig 3C and 3D). However, the increased PKA activity at the neurite tips was significantly decreased when endogenous GPR3 expression was down-regulated by the GPR3 siRNA. The down-regulation of PKA by the GPR3 siRNA was rescued by co-transfection of GPR3 siRNA and an siRNA-resistant GPR3 expression vector. We performed similar experiments using the SH-SY5Y neuroblastoma cell line. Real-time PCR analysis revealed that GPR3 was endogenously expressed in the SH-SY5Y cells, but GPR6 and GPR12 were expressed at very low levels in these cells (S3A Fig). The increased PKA activity at the neurite tips was also observed when GPR3 was up-regulated in these cells (S3C Fig). The PKA activity at the neurite tips was significantly reduced in the GPR3 siRNA-transfected cells compared to the control siRNA-transfected cells (S3D Fig). In addition, the GPR3 siRNA-mediated reduction of the PKA activity in the neurites was rescued when the siRNA-resistant GPR3 expression vector was co-transfected with GPR3 siRNA. All of these results indicated that GPR3 expression is associated with local PKA activity at the neurite tips in neuronal cells.

### Analyses of local GPR3 dynamics in CGNs

**Fig 4:**
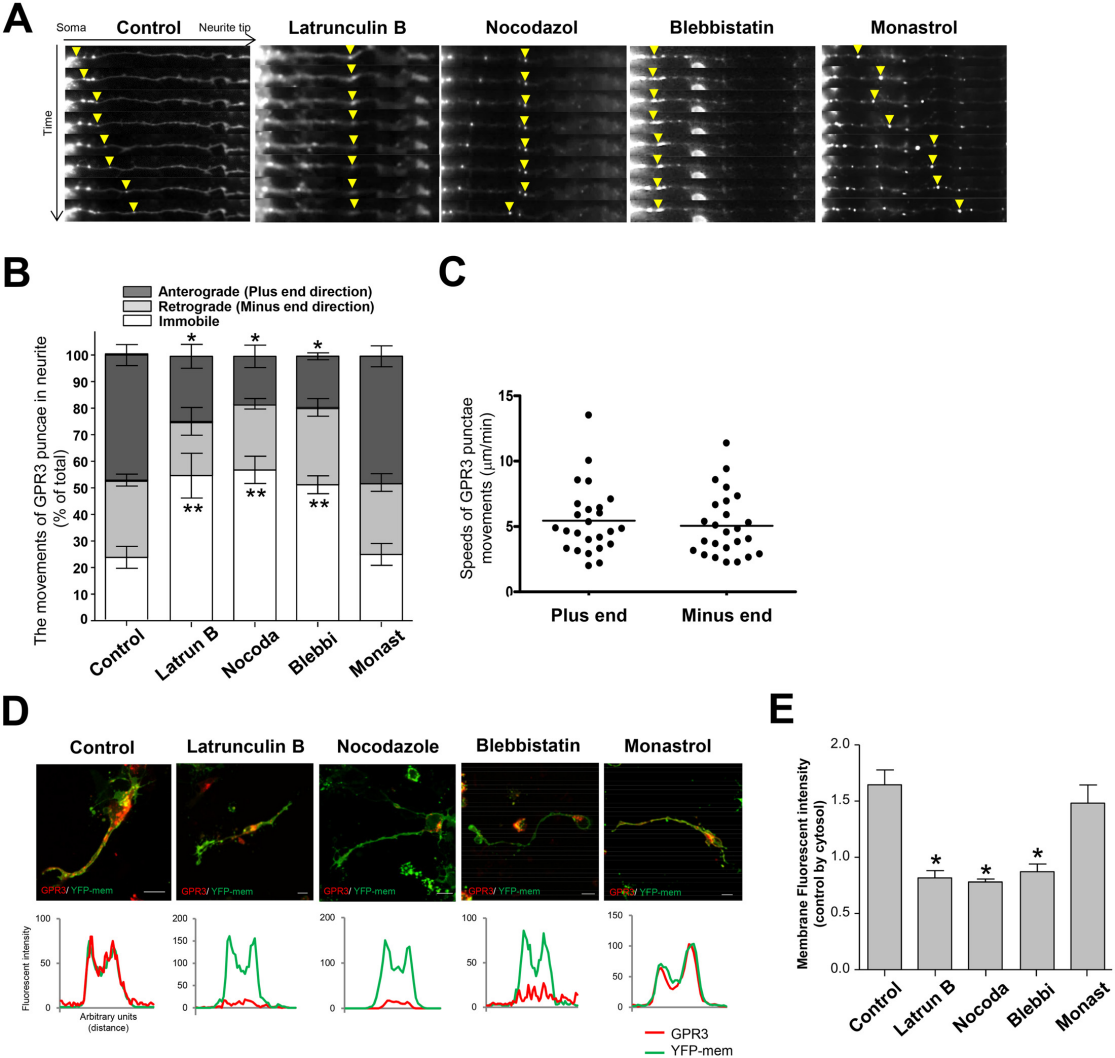
Fluorescence time-lapse imaging of a single GPR3-HT-expressing CGN. (A-B) The CGNs were transfected with the pGPR3-HT expression vector and were plated onto 0.03% PEI-coated plates. Forty-five hours after transfection, GPR3-HT was labeled with the TMR HaloTag ligand for 15 min. After three sequential washes in PBS, the cells were incubated with various inhibitors for another two hours. The cells were treated with 1 μM Latrunculin B, 3 μM Nocodazole, 50 μM Blebbistatin, or 2 mM Monastrol in the culture medium, respectively. As a control, a 1:1000 dilution of DMSO was added to the culture medium. The pGPR3-HT fluorescence in the CGNs was captured every 10 minutes using a fluorescent microscope. (A) Representative images of the CGNs from each condition were shown. The arrow indicates the GPR3 fluorescent puncta at each time point. Serial images were shown every 20 min. (B) The directions that the GPR3 puncta moved along the neurite were analyzed in each condition. The directions of puncta movement were divided into three groups: the plus end movement group, the minus end movement group, and the immobile group. The values were then expressed as the percentage of the number of puncta in each direction out of the total numbers of puncta. The numbers of puncta in plus end direction were significantly decreased when Latrunculin B, Nocodazole, Blebbistatin were included in the medium. The data represent the means ± SEM for each condition (n = 16). The asterisk (*) represents p < 0.0001 and the double asterisk (**) represents p < 0.05. (C) The speed of the GPR3 puncta were calculated from the time-lapse images. The mean speed of the GPR3 puncta in plus-end and minus-end directions were separately shown (n = 25). (D-E) The fluorescent intensity of GPR3 in the membranes at the neurite tips were evaluated with or without addition of various actin and tubulin inhibitors. The CGNs were co-transfected with pGPR3-HT and pYFP-mem. Twenty-four hours after transfection, some groups were treated with inhibitors as described above. Forty-six hours after transfection, the cells were labeled with the TMR HaloTag ligand for 2 hours. The cells were then fixed and the fluorescent images of GPR3-HT and YFP-mem were captured using a fluorescent microscope from the cells in each condition. The fluorescent intensity of GPR3 in the membranes at the neurite tips were evaluated by the line profiling method (detailed in the Materials and methods). (D) Representative images of the CGNs and the line profiling analyses from each condition were shown. (E) The fluorescent intensities of GPR3-HT in the plasma membrane and cytosol at the neurite tips were compared for each condition. The values were then expressed as the ratio of the fluorescent intensity at the plasma membrane to the cytosol at the neurite tips. The data represent the means ± SEM for each condition (n = 7). The asterisk (*) represents p < 0.0001.

Previous results suggested that the local GPR3-mediated PKA activation was associated with the local GPR3 dynamics. To address this issue, the temporal intracellular movements of the fluorescently tagged GPR3 were analyzed using fluorescent time-lapse microscopy. Surprisingly, the GPR3 fluorescent puncta were transported along the neurite in both directions over time (Fig 4A and 4B; S1 Movie). Statistical analysis revealed that approximately 50% of the GPR3 puncta was anterogradely transported toward the neurite tips toward the plus-end, whereas approximately 30% of the GPR3 puncta was retrogradely transported toward the minus-end. Fast and slow axonal transport have been reported [31,32]. The GPR3 puncta moved toward the plus-end at a mean rate of 5.5 ± 0.52 μm/min (n = 25), whereas they moved toward the minus-end at a mean rate of 5.1 ± 0.48 μm/min (n = 25), both of which were relatively slow types of axonal transportation (Fig 4C). It has been reported that molecular motor superfamily proteins, such as kinesin, dynein, and myosin, have played significant roles in numerous neuronal functions by transporting cargo [13]. We then speculated that GPR3 is transported via molecular motors to be distributed to local areas. To address this, the movements of the fluorescently tagged GPR3 were observed in cells that had been treated with various actin- or tubulin-dependent inhibitors. When the actin polymerization inhibitor Latrunculin B was administered to the GPR3-GFP-transfected CGNs, the plus-end movements of the GPR3 puncta were significantly inhibited (Fig 4A and 4B). The same trend of inhibition was observed when microtubule polymerization inhibitor Nocodazole was administered to the GPR3-GFP-transfected CGNs. We then asked whether the transport of the GPR3 puncta is modulated by a myosin II inhibitor. Administration of the myosin II-specific inhibitor Blebbistatin also abrogated the time-dependent plus-end movements of the GPR3 puncta. However, administration of Monastrol, which is a potential kinesin-5 inhibitor, did not disturb the movements of GPR3. The minus-end movements of the GPR3 puncta were not altered by the actin or tubulin inhibitors. Therefore, these results indicated that the movements of the GPR3 puncta along the CGN neurites are mediated by the actin- and/or tubulin-dependent cargo transport systems, particularly the myosin II-dependent system.

Next, we asked whether the transportation of the GPR3 puncta in neurite is associated with the local expression of GPR3. To address this, we transfected the fluorescently tagged GPR3 expression vector and evaluated the fluorescent intensity of GPR3 at the neurite tips with or without the actin or tubulin inhibitors. When Latrunculin B or Nocodazole was administered to these cells, the fluorescent intensity of GPR3 in the plasma membrane at the neurite tips was remarkably decreased compared to the control (Fig 4D and 4E). Furthermore, administration of Blebbistatin also decreased the fluorescent intensity of GPR3 at the neurite tips. However, the fluorescent intensity of GPR3 was not changed when Monastrol was administered. Thus, these results indicated that the GPR3 puncta are preferentially transported toward at the neurite tips through actin- and tubulin-dependent mechanisms.

### GPR3 transport in neurites was associated with local elevation of PKA activity at the neurite tips

**Fig 5:**
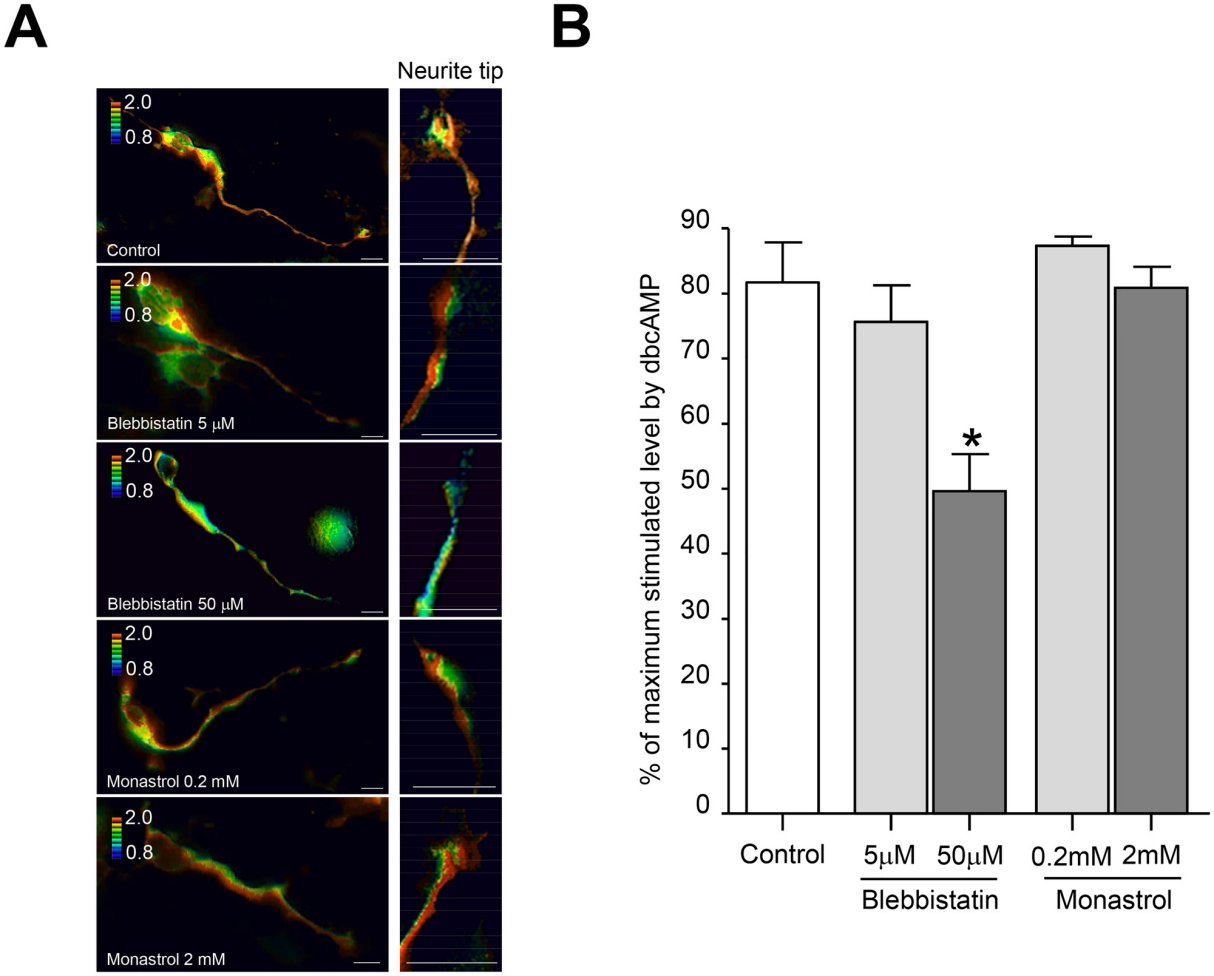
The PKA activity at the neurite tips was modulated by the local transport of GPR3 in CGNs. (A-B) Forty-five hours after transfection, GPR3-HT was labeled with the TMR HaloTag ligand for 15 min. After three sequential washes in PBS, the cells were incubated with Blebbistatin or Monastrol for another 2 hours. As a control, a 1:1000 dilution of DMSO was added to the culture medium. After capturing the CFP and FRET images, 1 mM dbcAMP was applied to the bath and additional CFP and FRET images were captured. (A) The representative FRET/CFP images from control, Blebbistatin-treated (5 μM, 50 μM), and Monastrol-treated (0.2 mM, 2 mM) CGNs at the neurite tips were shown. (B) The FRET/CFP ratio in each inhibitor-treated group were analyzed identically. The FRET/CFP ratios are expressed as the percentage of the maximum level stimulated by 1 mM dbcAMP in each cell. The data represent the means ± SEM for each condition (n = 8). The asterisk (*) represents p < 0.005.

Finally, we asked whether the movements of GPR3 in neurites affect the local PKA activity. To address this, GPR3- and AKAR3-EV-expressing CGNs were treated with the myosin II inhibitor. Administration of Blebbistatin (50 μM) significantly decreased the levels of PKA at the neurite tips, whereas addition of Monastrol did not change the PKA levels (Fig 5A and 5B). These results implicated that the GPR3 puncta were transported to the plasma membrane at the neurite tips in myosin II-dependent manner, thereby contributing to the local activation of PKA at the neurite tips.

## Discussion

In the present study, we demonstrate the subcellular localization of GPR3 in CGNs for the first time. Time-lapse experiments of the GPR3-transfected CGNs revealed that GPR3 was transported along the neurite and was predominantly distributed at the neurite tips. The movements of the GPR3 puncta were highly correlated with local PKA activation at the neurite tips.

We performed X-gal staining in GPR3 knockout, LacZ knock-in mice to assess the distribution of GPR3 in the mouse brain because there is no good mouse GPR3 antibody available for immunohistochemistry. We have previously reported that GPR3 is highly expressed in the medial habenular nucleus, cerebral cortex, hippocampus, olfactory bulb, striatum, and cerebellum [2,5]. In addition to these areas, we have clarified additional areas of GPR3 expression in the thalamus, pons, and spinal cord. Because X-gal staining of the GPR3 knockout, LacZ knock-in mouse only represents the GPR3 promoter activity, we could not evaluate the expression levels of GPR3 in each area. Further examinations, such as utilizing a GPR3-GFP knock-in mouse, will clarify the more precise distribution of GPR3 in the rodent brain. Recently, it has been reported that monoamine neurotransmitter levels in the hypothalamus, frontal cortex, hippocampus are decreased in the GPR3 knockout mice, which was significantly correlated to emotional disorders using several behavior analyses [8]. Moreover, our present report suggested that the expression of GPR3 in neurons contributed to the basal activation of the ERK or Akt signaling pathways, thereby maintaining neuronal homeostasis and cell survival [4]. Thus, neuronal expression of GPR3 may potentially regulate various neuronal functions; however, the role of GPR3 in the central nervous system remains to be elucidated.

In the present study, transfection of fluorescently tagged GPR3 revealed that GPR3 is distributed along the plasma membrane and in the Golgi body and endosomes in CGNs. In addition, PKA activation by GPR3 was limited along the plasma membrane, as determined by the PKA FRET sensor. The membrane expression of GPR3 was suggested to be internalized by overexpression of GRK2 and beta-arrestin2 via phosphorylation at serine/threonine residues of GPR3 in HEK293 cells [12]. Moreover, they have indicated that the plasma membrane expression of GPR3 functionally activates the cAMP levels in these cells, as determined by an Epac-based cAMP FRET sensor; the GPR3-mediated increases in cAMP levels are lost when GPR3 is internalized. These results are consistent with our current results in CGNs, because the plasma membrane expression of GPR3 functionally activates PKA in adjacent areas. However, a recent report on a related GPCR suggested that the Gαs protein of the β2-adrenergic receptor was activated not only in the plasma membrane but also in the early endosome membrane [33]. Because GPR3 is currently an orphan receptor, we could not determine the ligand-dependent PKA activity in endosomes.

In this study, we clarified that GPR3 puncta were transported toward the neurite tips, thereby contributing to the local activation of PKA. In our previous paper, we have revealed that the expression of GPR3 in CGNs promoted the activation of the ERK and Akt signaling pathways at physiological level, thereby contributing to the neuronal survival [4]. These results indicated that the downstream signaling pathways activated by GPR3 might mediate various diverse functions in neurons. However, it remains to be understood how GPR3 regulates these downstream signaling pathways to exert neuronal functions without addition of any ligands. The similar constitutive active feature has been also reported in other GPRs including histamine H3 receptor, 5-hydroxytryptamine 4 (5-HT_4_) receptor, and a third-internal loop mutant of the β2-adrenergic receptor [34–36]. In addition, the third intracellular loop and the C-terminal tail, which are important for the coupling of GPR to G-proteins, are associated with constitutive features of these GPRs. Indeed, a third-internal loop mutant of GPR3 induced elevated basal level of intracellular cAMP compared with wild-type GPR3 in HEK293 cells [12]. Moreover, the constitutive active features of these GPRs are frequently abrogated by inverse agonists. Considering that the membrane expression of GPR3 is internalized by overexpression of GRK2 and beta-arrestin2, the unknown agonists or inverse agonists might modulate constitutive active feature of GPR3 in neurons. Besides, the activities of GPR3 might be also regulated by the amount of expression or the areas of expression where GPR3 puncta are transported. The future deorphanization of GPR3 and clarifying its local dynamics may provide a clearer understanding of GPR3 neuronal expression and how it functionally activates the downstream signaling pathways.

In the current study, the GPR3 puncta were mainly transported toward the neurite tips in the CGNs. Fast and slow axonal transport has been reported [31,32]. Membranous organelles and Golgi-derived vesicles were rapidly transported along microtubules and microfilaments using molecular motor proteins, such as kinesins and dyneins. However, slow transport is observed in axons, which is divided into two groups: slow component b (SCb) and slow component a (SCa). SCa, which includes tubulin, spectrin and the tau proteins, transports microfilaments and neurofilaments at the slowest speed (0.002–0.01 μm/s). Similarly, SCb transports microfilaments and cytosolic protein complexes with the next slowest speed via unknown mechanisms. Interestingly, in developing hippocampal neurons, the anterograde wave-like movements of growth cone-like structures are observed along the axons and dendrites [37,38]. The wave-like structure contains actin and associated proteins, which is consistent with the SCb components F-actin, clathrin, GAP-43, and ezrin. Therefore, the wave-like structure is thought to be a slower system of bulk transport in the developing axon. The mechanisms underlying the wave-like structure-mediated axonal transport have not been identified; however, anterograde transports of the SCb components are inhibited by both actin and microtubule inhibitors [37,38]. In the present study, the movement of the GPR3 puncta was relatively slow and was similar to the “waves” or SCb. In addition, wave-like structures were also observed in developing CGNs, and most of the GPR3 puncta in the neurites were distributed in the wave-like structures (data not shown). Furthermore, the movements of the GPR3 puncta were completely abrogated not only by actin-myosin inhibitors but also by microtubule inhibitors, which are correlated with the wave-mediated transport. Thus, these results implicated that the GPR3 puncta may be categorized as an SCb component and anterogradely transported with the wave-like-structures in the developing CGNs.

In addition to actin or microtubule polymerization inhibitors, the movements of the GPR3 puncta were also completely abrogated by the administration of the Myosin II inhibitor, blebbistatin. In CGNs, myosin II and F-actin are concentrated near the centrosome and the leading process, whereas the microtubules are abundantly distributed in the neurite shafts [23]. Both actin and microtubules have coordinated roles in neuronal migration and synapse function [23,39]. Actin filaments interacted with microtubules at the growth cone neck, and microtubule-mediated transport is inhibited by a myosin II inhibitor [40]. In addition, Shootin1, which is expressed in developing hippocampal neurons, is also anterogradely transported to the growth cones using wave-like structures in a myosin II-dependent manner [41]. Based on these reports and the current findings, we suggested that the actin-dependent and microtubule-dependent transport systems are associated with each other, and may play a role in the myosin II-dependent transport of SCb components along the neurite. Further investigations will be needed to clarify the mechanisms underlying the myosin II-dependent transport of GPR3.

In the current study, we found that the expression of GPR3 in developing CGNs correlated with local PKA activation at the neurite tips in a myosin II-dependent manner. In the development of cultured hippocampal neurons, several minor processes are elaborated from the cell body, and one of the minor processes extends rapidly to form the axon [42,43]. Similar polarization is observed in the development of CGNs [44]. Recent studies suggested that several internal cues (i.e., Rac1, MARK2 kinase, SAD kinase, and CAM Kinase) and external cues (i.e., neurotrophins, Wnts, IGF-1, TGF-β, and TAG-1) affect axon formation [45]. Among them, SAD kinase is phosphorylated by LKB1, which is phosphorylated by PKA [46,47]. Thus, the downstream pathway of cAMP is suggested to be a key regulator of neuronal polarization [48]. The expression of GPR3 in CGNs is increased during neuronal differentiation and maintained thereafter [5]. In the current study, we found that GPR3 was transported to and accumulated at increased levels at the neurite tips and thus contributed to local PKA activation at the neurite tips in CGN development. Together, these results suggested that the GPR3-mediated local PKA activation may play a role in axon formation during neuronal development.

## Supporting Information

S1 Fig The distribution of GPR3 promoter activity in the mouse central nervous system. A GPR3-/-; LacZ +/+ mouse was employed to determine the distribution of GPR3 in the developing postnatal cerebellum, where the E. coli LacZ gene was substituted into the GPR3 locus. Coronal or sagittal sections of the central nervous system were cut with a vibratome at 100 μm thickness. The GPR3 promoter activity was evaluated by β-galactosidase expression in the slices using X-gal staining (detailed in the Materials and methods). The dark green staining in each section represented the locations where the GPR3 promoter was activated. A: anterior from the bregma; P: posterior from the bregma. Scale bar = 0.5 mm.
(TIF)

S2 Fig The acceptor photo bleaching experiments for AKAR3-EV and the bleedthrough corrections. (A) We performed acceptor photo bleaching experiments to confirm that the FRET phenomena of AKAR3-EV has really occurred in our system. CGNs were transfected with AKAR3EV expression vector. Forty eight hours after transfection, 1mM dbcAMP was administrated in the medium and incubated for 15 min. FRET and CFP images were taken every one min. and acceptor photo bleaching was performed by exposing YFP excitation light for one min without using a natural density filter nor diffusion filter. After bleaching, FRET and CFP images were also taken every one min. (B-C) To address CFP bleedthrough into YFP channel, CFP only expressing vector was transfected in CGNs. CFP transfected cells were then excited by 438nm CFP excitation light and fluorescence was recorded in the CFP and YFP emission channel. YFP channel intensity per CFP channel intensity was calculated and found out 0.23 in our system (Coefficient α). To address YFP excitation by CFP excitation light, YFP only expressing vector was transfected in CGNs. YFP transfected cells were then excited by CFP or YFP excitation light and fluorescence was recorded in the YFP emission channel. YFP channel intensity excited by CFP emission per FRET channel intensity excited by YFP emission was calculated and found out 0.01 in our system (Coefficient β). We then calculated corrected FRET values according to the following equation by MetaMorph software. [CorrFRET] = [RawFRET] − β*[Acceptor] − α*[Donor].
(TIF)

S3 Fig The PKA activity at the neurite tips was modulated by the expression of GPR3 in SH-SY5Y cells. (A) We evaluated the intrinsic expression of GPR3 in SH-SY5Y cells under normal culture conditions. The total RNA was extracted from SH-SY5Y cells in normal culture conditions. The RNA samples were subjected to quantitative RT-PCR analysis using primers specific to human GPR3, GPR6, and GPR12. All quantitative data were adjusted using the levels of the GAPDH mRNA as internal control. Real-time PCR analysis revealed that GPR3 was endogenously expressed in SH-SY5Y cells, but GPR6 and GPR12 were expressed at very low levels in these cells. (B) The SH-SY5Y cells were transfected with a control siRNA or GPR3 siRNA. Twenty-four hours post-transfection, the levels of the GPR3 mRNA was examined by real-time PCR. The endogenous expression of GPR3 was reduced to ~10–15% by the transfection of the GPR3 siRNA. (C) SH-SY5Y cells were co-transfected with AKAR3-EV and a GPR3 expression plasmid. Forty-eight hours after transfection, the FRET/CFP images were captured using a fluorescent microscope. After capturing the images, some cells were treated with 1 mM dbcAMP for 15 min and additional FRET/CFP images were captured to evaluate the fully activated PKA. The FRET/CFP ratio in each group were analyzed identically in each dish, as previously described. The FRET/CFP ratios are expressed as the percentage of the maximum level stimulated by 1 mM dbcAMP in each cell. The data represent the means ± SEM for each condition (n = 8). The asterisk (*) represents p < 0.0001. (D) SH-SY5Y cells were co-transfected with AKAR3-EV and the GPR3 siRNA. Forty-eight hours after transfection, the FRET/CFP images were captured using a fluorescent microscope. For the rescue experiments, a GPR3-expressing plasmid was also co-transfected with the GPR3 siRNA. The FRET value in each condition was evaluated as described above. The data represent the means ± SEM for each condition (n = 6). The asterisk (*) represents p < 0.005.
(TIF)

S1 Movie Fluorescence time-lapse movie of a single GPR3-HT-expressing CGN. Representative movie of the GPR3-HT transfected CGNs was shown. The time counter in the upper left corner represents minutes after initiation of recording.
(AVI)

## References

[1] Eggerickx D, Denef JF, Labbe O, Hayashi Y, Refetoff S, Vassart G, et al Molecular cloning of an orphan G-protein-coupled receptor that constitutively activates adenylate cyclase. Biochem J. 1995;309 (Pt 3):837–43. Epub 1995/08/01. . 7639700 10.1042/bj3090837 PMC1135708

[2] Saeki Y, Ueno S, Mizuno R, Nishimura T, Fujimura H, Nagai Y, et al Molecular cloning of a novel putative G protein-coupled receptor (GPCR21) which is expressed predominantly in mouse central nervous system. FEBS Lett. 1993;336(2):317–22. Epub 1993/12/27. 0014-5793(93)80828-I [pii]. . 8262253 10.1016/0014-5793(93)80828-i

[3] Tanaka S, Ishii K, Kasai K, Yoon SO, Saeki Y. Neural expression of G protein-coupled receptors GPR3, GPR6, and GPR12 up-regulates cyclic AMP levels and promotes neurite outgrowth. J Biol Chem. 2007;282(14):10506–15. Epub 2007/02/08. M700911200 [pii] 10.1074/jbc.M700911200 . 17284443

[4] Tanaka S, Miyagi T, Dohi E, Seki T, Hide I, Sotomaru Y, et al Developmental expression of GPR3 in rodent cerebellar granule neurons is associated with cell survival and protects neurons from various apoptotic stimuli. Neurobiol Dis. 2014;68:215–27. Epub 2014/04/29. S0969-9961(14)00096-5 [pii] 10.1016/j.nbd.2014.04.007 . 24769160

[5] Tanaka S, Shaikh IM, Chiocca EA, Saeki Y. The Gs-linked receptor GPR3 inhibits the proliferation of cerebellar granule cells during postnatal development. PLoS One. 2009;4(6):e5922 Epub 2009/06/16. 10.1371/journal.pone.0005922 . 19526062 PMC2691605

[6] Thathiah A, Horre K, Snellinx A, Vandewyer E, Huang Y, Ciesielska M, et al beta-arrestin 2 regulates Abeta generation and gamma-secretase activity in Alzheimer's disease. Nat Med. 2013;19(1):43–9. Epub 2012/12/04. nm.3023 [pii] 10.1038/nm.3023 . 23202293

[7] Thathiah A, Spittaels K, Hoffmann M, Staes M, Cohen A, Horre K, et al The orphan G protein-coupled receptor 3 modulates amyloid-beta peptide generation in neurons. Science. 2009;323(5916):946–51. Epub 2009/02/14. 323/5916/946 [pii] 10.1126/science.1160649 . 19213921

[8] Valverde O, Celerier E, Baranyi M, Vanderhaeghen P, Maldonado R, Sperlagh B, et al GPR3 receptor, a novel actor in the emotional-like responses. PLoS One. 2009;4(3):e4704 Epub 2009/03/05. 10.1371/journal.pone.0004704 . 19259266 PMC2649507

[9] Ruiz-Medina J, Ledent C, Valverde O. GPR3 orphan receptor is involved in neuropathic pain after peripheral nerve injury and regulates morphine-induced antinociception. Neuropharmacology. 2011;61(1–2):43–50. Epub 2011/03/01. S0028-3908(11)00084-0 [pii] 10.1016/j.neuropharm.2011.02.014 . 21352831

[10] Tourino C, Valjent E, Ruiz-Medina J, Herve D, Ledent C, Valverde O. The orphan receptor GPR3 modulates the early phases of cocaine reinforcement. Br J Pharmacol. 2012;167(4):892–904. Epub 2012/05/23. 10.1111/j.1476-5381.2012.02043.x . 22612385 PMC3575787

[11] Mehlmann LM, Saeki Y, Tanaka S, Brennan TJ, Evsikov AV, Pendola FL, et al The Gs-linked receptor GPR3 maintains meiotic arrest in mammalian oocytes. Science. 2004;306(5703):1947–50. Epub 2004/12/14. 306/5703/1947 [pii] 10.1126/science.1103974 . 15591206

[12] Lowther KM, Uliasz TF, Gotz KR, Nikolaev VO, Mehlmann LM. Regulation of Constitutive GPR3 Signaling and Surface Localization by GRK2 and beta-arrestin-2 Overexpression in HEK293 Cells. PLoS One. 2013;8(6):e65365 Epub 2013/07/05. 10.1371/journal.pone.0065365 PONE-D-12-38312 [pii]. . 23826079 PMC3694969

[13] Hirokawa N, Niwa S, Tanaka Y. Molecular motors in neurons: transport mechanisms and roles in brain function, development, and disease. Neuron. 2010;68(4):610–38. Epub 2010/11/26. S0896-6273(10)00781-6 [pii] 10.1016/j.neuron.2010.09.039 . 21092854

[14] Niwa S, Tanaka Y, Hirokawa N. KIF1Bbeta- and KIF1A-mediated axonal transport of presynaptic regulator Rab3 occurs in a GTP-dependent manner through DENN/MADD. Nat Cell Biol. 2008;10(11):1269–79. Epub 2008/10/14. ncb1785 [pii] 10.1038/ncb1785 . 18849981

[15] Arimura N, Kimura T, Nakamuta S, Taya S, Funahashi Y, Hattori A, et al Anterograde transport of TrkB in axons is mediated by direct interaction with Slp1 and Rab27. Dev Cell. 2009;16(5):675–86. Epub 2009/05/23. S1534-5807(09)00095-1 [pii] 10.1016/j.devcel.2009.03.005 . 19460344

[16] Setou M, Seog DH, Tanaka Y, Kanai Y, Takei Y, Kawagishi M, et al Glutamate-receptor-interacting protein GRIP1 directly steers kinesin to dendrites. Nature. 2002;417(6884):83–7. Epub 2002/05/03. 10.1038/nature743 nature743 [pii]. . 11986669

[17] Twelvetrees AE, Yuen EY, Arancibia-Carcamo IL, MacAskill AF, Rostaing P, Lumb MJ, et al Delivery of GABAARs to synapses is mediated by HAP1-KIF5 and disrupted by mutant huntingtin. Neuron. 2010;65(1):53–65. Epub 2010/02/16. S0896-6273(09)00997-0 [pii] 10.1016/j.neuron.2009.12.007 . 20152113 PMC2841506

[18] Tanaka Y, Kanai Y, Okada Y, Nonaka S, Takeda S, Harada A, et al Targeted disruption of mouse conventional kinesin heavy chain, kif5B, results in abnormal perinuclear clustering of mitochondria. Cell. 1998;93(7):1147–58. Epub 1998/07/10. S0092-8674(00)81459-2 [pii]. . 9657148 10.1016/s0092-8674(00)81459-2

[19] Kamal A, Almenar-Queralt A, LeBlanc JF, Roberts EA, Goldstein LS. Kinesin-mediated axonal transport of a membrane compartment containing beta-secretase and presenilin-1 requires APP. Nature. 2001;414(6864):643–8. Epub 2001/12/12. 414643a [pii]. . 11740561 10.1038/414643a

[20] Jacobson C, Schnapp B, Banker GA. A change in the selective translocation of the Kinesin-1 motor domain marks the initial specification of the axon. Neuron. 2006;49(6):797–804. Epub 2006/03/18. S0896-6273(06)00095-X [pii] 10.1016/j.neuron.2006.02.005 . 16543128

[21] Ohashi S, Koike K, Omori A, Ichinose S, Ohara S, Kobayashi S, et al Identification of mRNA/protein (mRNP) complexes containing Puralpha, mStaufen, fragile X protein, and myosin Va and their association with rough endoplasmic reticulum equipped with a kinesin motor. J Biol Chem. 2002;277(40):37804–10. Epub 2002/07/31. 10.1074/jbc.M203608200 M203608200 [pii]. . 12147688

[22] Yano H, Ninan I, Zhang H, Milner TA, Arancio O, Chao MV. BDNF-mediated neurotransmission relies upon a myosin VI motor complex. Nat Neurosci. 2006;9(8):1009–18. Epub 2006/07/05. nn1730 [pii] 10.1038/nn1730 . 16819522

[23] Solecki DJ, Trivedi N, Govek EE, Kerekes RA, Gleason SS, Hatten ME. Myosin II motors and F-actin dynamics drive the coordinated movement of the centrosome and soma during CNS glial-guided neuronal migration. Neuron. 2009;63(1):63–80. Epub 2009/07/18. S0896-6273(09)00435-8 [pii] 10.1016/j.neuron.2009.05.028 . 19607793 PMC2737100

[24] Vallee RB, Seale GE, Tsai JW. Emerging roles for myosin II and cytoplasmic dynein in migrating neurons and growth cones. Trends Cell Biol. 2009;19(7):347–55. Epub 2009/06/16. S0962-8924(09)00099-3 [pii] 10.1016/j.tcb.2009.03.009 . 19524440 PMC2844727

[25] Wang D, She L, Sui YN, Yuan XB, Wen Y, Poo MM. Forward transport of proteins in the plasma membrane of migrating cerebellar granule cells. Proc Natl Acad Sci U S A. 2012;109(51):E3558–67. Epub 2012/12/06. 1219203110 [pii] 10.1073/pnas.1219203110 . 23213239 PMC3529063

[26] Komatsu N, Aoki K, Yamada M, Yukinaga H, Fujita Y, Kamioka Y, et al Development of an optimized backbone of FRET biosensors for kinases and GTPases. Mol Biol Cell. 2011;22(23):4647–56. Epub 2011/10/07. mbc.E11-01-0072 [pii] 10.1091/mbc.E11-01-0072 . 21976697 PMC3226481

[27] Borner S, Schwede F, Schlipp A, Berisha F, Calebiro D, Lohse MJ, et al FRET measurements of intracellular cAMP concentrations and cAMP analog permeability in intact cells. Nature protocols. 2011;6(4):427–38. 10.1038/nprot.2010.198 . 21412271

[28] Spiering D, Bravo-Cordero JJ, Moshfegh Y, Miskolci V, Hodgson L. Quantitative ratiometric imaging of FRET-biosensors in living cells. Methods in cell biology. 2013;114:593–609. 23931524 10.1016/B978-0-12-407761-4.00025-7 PMC3789067

[29] Freudzon L, Norris RP, Hand AR, Tanaka S, Saeki Y, Jones TL, et al Regulation of meiotic prophase arrest in mouse oocytes by GPR3, a constitutive activator of the Gs G protein. J Cell Biol. 2005;171(2):255–65. Epub 2005/10/26. jcb.200506194 [pii] 10.1083/jcb.200506194 . 16247026 PMC2171177

[30] Verveer PJ, Rocks O, Harpur AG, Bastiaens PI. Imaging protein interactions by FRET microscopy: FRET measurements by acceptor photobleaching. CSH protocols. 2006;2006(6). 10.1101/pdb.prot4598 . 22485985

[31] Brown A. Axonal transport of membranous and nonmembranous cargoes: a unified perspective. J Cell Biol. 2003;160(6):817–21. Epub 2003/03/19. 10.1083/jcb.200212017 12642609 PMC2173776

[32] Lasek RJ, Garner JA, Brady ST. Axonal transport of the cytoplasmic matrix. J Cell Biol. 1984;99(1 Pt 2):212s–21s. Epub 1984/07/01. 6378920 10.1083/jcb.99.1.212s PMC2275578

[33] Irannejad R, Tomshine JC, Tomshine JR, Chevalier M, Mahoney JP, Steyaert J, et al Conformational biosensors reveal GPCR signalling from endosomes. Nature. 2013;495(7442):534–8. Epub 2013/03/22. 10.1038/nature12000 23515162 PMC3835555

[34] Claeysen S, Sebben M, Becamel C, Bockaert J, Dumuis A. Novel brain-specific 5-HT4 receptor splice variants show marked constitutive activity: role of the C-terminal intracellular domain. Molecular pharmacology. 1999;55(5):910–20. . 10220570

[35] Lefkowitz RJ, Cotecchia S, Samama P, Costa T. Constitutive activity of receptors coupled to guanine nucleotide regulatory proteins. Trends in pharmacological sciences. 1993;14(8):303–7. 10.1016/0165-6147(93)90048-O . 8249148

[36] Morisset S, Rouleau A, Ligneau X, Gbahou F, Tardivel-Lacombe J, Stark H, et al High constitutive activity of native H3 receptors regulates histamine neurons in brain. Nature. 2000;408(6814):860–4. 10.1038/35048583 . 11130725

[37] Ruthel G, Banker G. Actin-dependent anterograde movement of growth-cone-like structures along growing hippocampal axons: a novel form of axonal transport? Cell motility and the cytoskeleton. 1998;40(2):160–73. Epub 1998/06/20. 10.1002/(sici)1097-0169(1998)40:2<160::aid-cm5>3.0.co;2-j . 9634213

[38] Ruthel G, Banker G. Role of moving growth cone-like "wave" structures in the outgrowth of cultured hippocampal axons and dendrites. Journal of neurobiology. 1999;39(1):97–106. Epub 1999/04/23. . 10213456 10.1002/(sici)1097-4695(199904)39:1<97::aid-neu8>3.0.co;2-z

[39] Kneussel M, Wagner W. Myosin motors at neuronal synapses: drivers of membrane transport and actin dynamics. Nat Rev Neurosci. 2013;14(4):233–47. Epub 2013/03/14. nrn3445 [pii] 10.1038/nrn3445 . 23481482

[40] Burnette DT, Ji L, Schaefer AW, Medeiros NA, Danuser G, Forscher P. Myosin II activity facilitates microtubule bundling in the neuronal growth cone neck. Dev Cell. 2008;15(1):163–9. Epub 2008/07/09. 10.1016/j.devcel.2008.05.016 18606149 PMC2548298

[41] Toriyama M, Shimada T, Kim KB, Mitsuba M, Nomura E, Katsuta K, et al Shootin1: A protein involved in the organization of an asymmetric signal for neuronal polarization. J Cell Biol. 2006;175(1):147–57. Epub 2006/10/13. 10.1083/jcb.200604160 17030985 PMC2064506

[42] Caceres A, Banker GA, Binder L. Immunocytochemical localization of tubulin and microtubule-associated protein 2 during the development of hippocampal neurons in culture. J Neurosci. 1986;6(3):714–22. Epub 1986/03/01. . 3514816 10.1523/JNEUROSCI.06-03-00714.1986 PMC6568475

[43] Dotti CG, Sullivan CA, Banker GA. The establishment of polarity by hippocampal neurons in culture. J Neurosci. 1988;8(4):1454–68. Epub 1988/04/01. . 3282038 10.1523/JNEUROSCI.08-04-01454.1988 PMC6569279

[44] Powell SK, Rivas RJ, Rodriguez-Boulan E, Hatten ME. Development of polarity in cerebellar granule neurons. Journal of neurobiology. 1997;32(2):223–36. Epub 1997/02/01. . 9032664 10.1002/(sici)1097-4695(199702)32:2<223::aid-neu7>3.0.co;2-a

[45] Funahashi Y, Namba T, Nakamuta S, Kaibuchi K. Neuronal polarization in vivo: Growing in a complex environment. Curr Opin Neurobiol. 2014;27C:215–23. Epub 2014/05/08. S0959-4388(14)00088-9 [pii] 10.1016/j.conb.2014.04.009 . 24800936

[46] Shelly M, Cancedda L, Heilshorn S, Sumbre G, Poo MM. LKB1/STRAD promotes axon initiation during neuronal polarization. Cell. 2007;129(3):565–77. Epub 2007/05/08. S0092-8674(07)00469-2 [pii] 10.1016/j.cell.2007.04.012 . 17482549

[47] Shelly M, Poo MM. Role of LKB1-SAD/MARK pathway in neuronal polarization. Dev Neurobiol. 2011;71(6):508–27. Epub 2011/03/19. 10.1002/dneu.20884 . 21416623

[48] Shelly M, Lim BK, Cancedda L, Heilshorn SC, Gao H, Poo MM. Local and long-range reciprocal regulation of cAMP and cGMP in axon/dendrite formation. Science. 2010;327(5965):547–52. Epub 2010/01/30. 327/5965/547 [pii] 10.1126/science.1179735 . 20110498

