# Supplementary figures and images for: The Subcellular Dynamics of the Gs-Linked Receptor GPR3 Contribute to the Local Activation of PKA in Cerebellar Granular Neurons
Source: PLoS One. 2016 Jan 22;11(1):e0147466. doi: 10.1371/journal.pone.0147466 (PMC4723318; doi:10.1371/journal.pone.0147466)

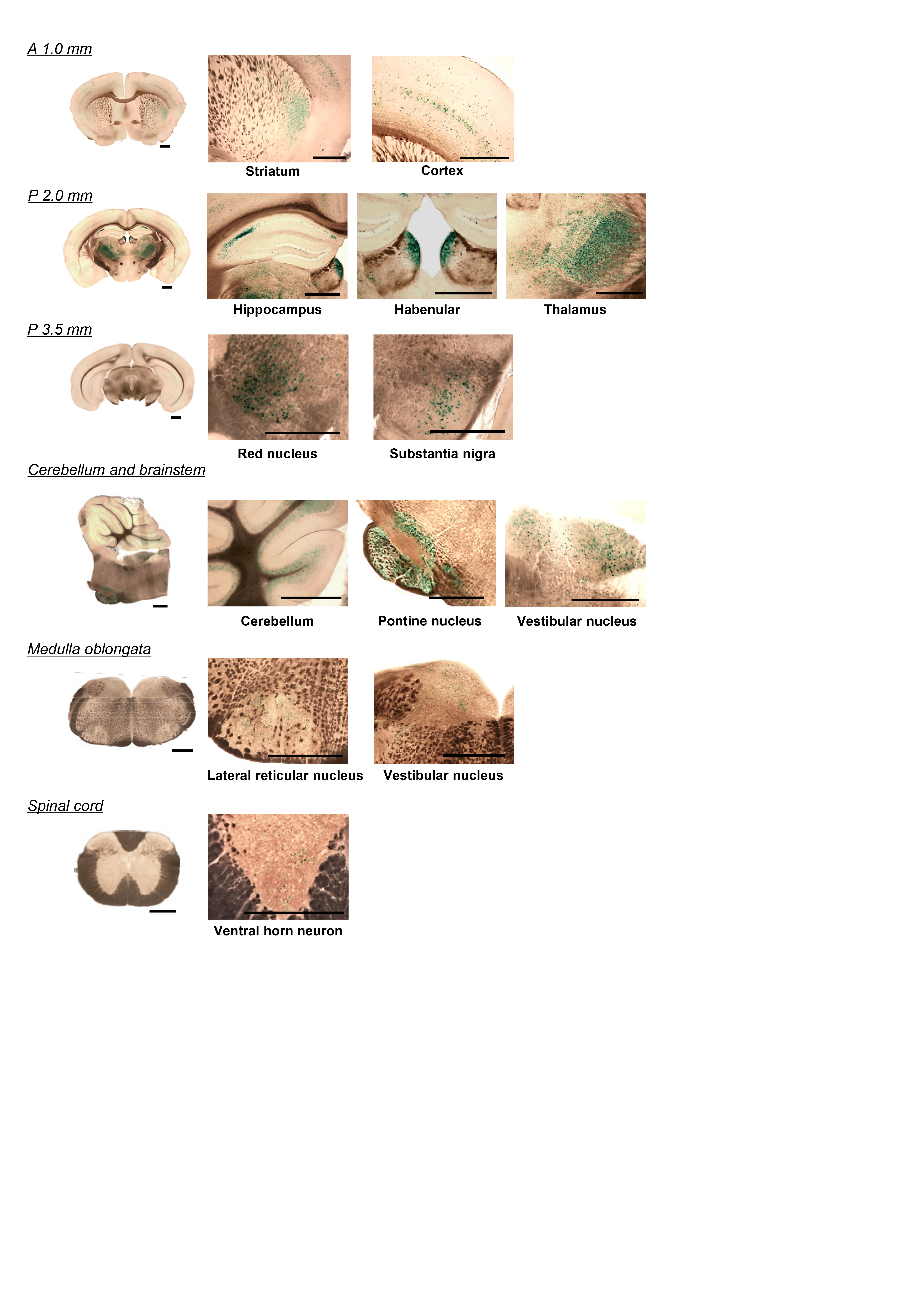

Supplement: S1 Fig — A GPR3-/-; LacZ +/+ mouse was employed to determine the distribution of GPR3 in the developing postnatal cerebellum, where the E. coli LacZ gene was substituted into the GPR3 locus. Coronal or sagittal sections of the central nervous system were cut with a vibratome at 100 μm thickness. The GPR3 promoter activity was evaluated by β-galactosidase expression in the slices using X-gal staining (detailed in the Materials and methods). The dark green staining in each section represented the locations where the GPR3 promoter was activated. A: anterior from the bregma; P: posterior from the bregma. Scale bar = 0.5 mm. (TIF) [file pone.0147466.s001.tif]

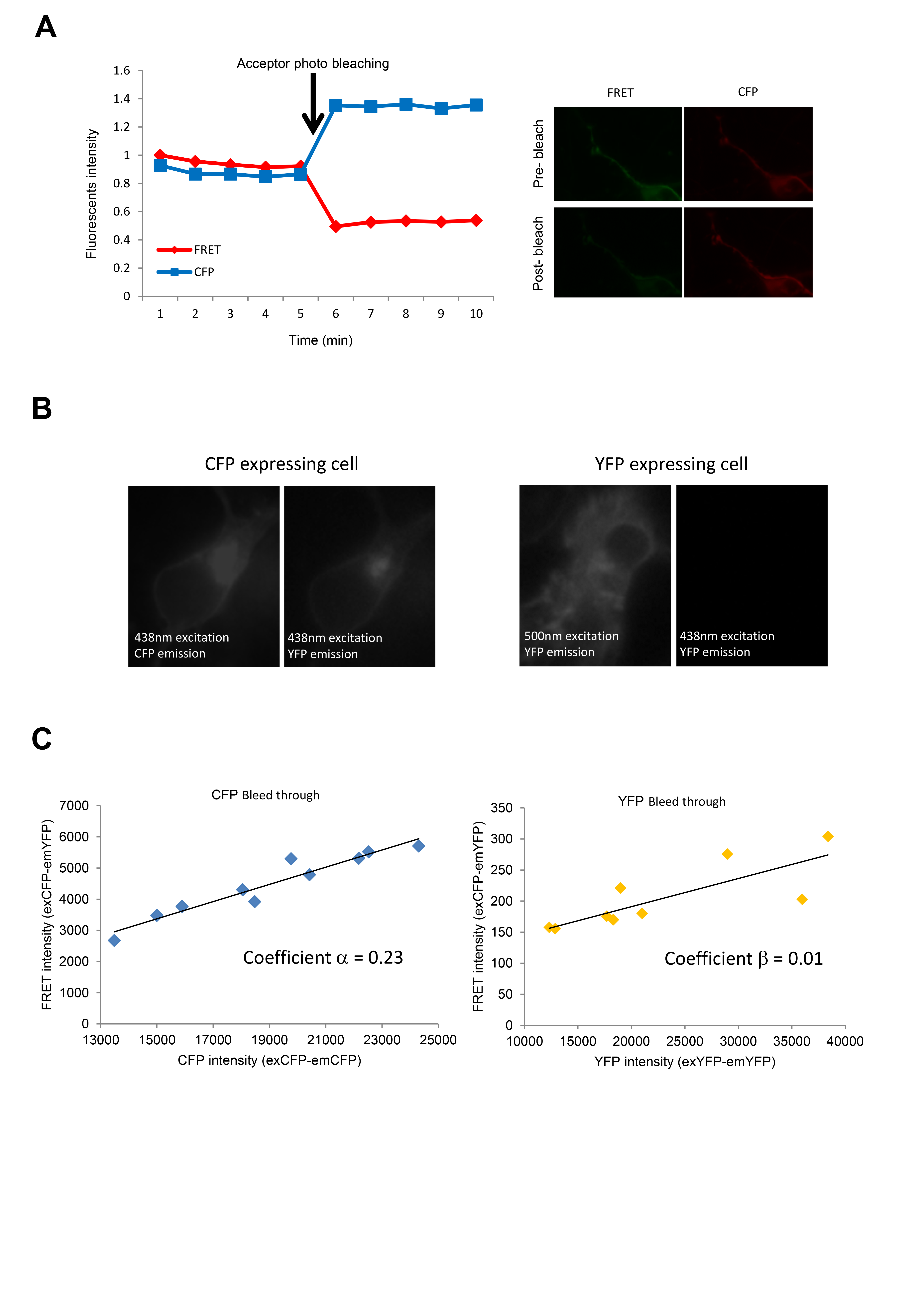

Supplement: S2 Fig — (A) We performed acceptor photo bleaching experiments to confirm that the FRET phenomena of AKAR3-EV has really occurred in our system. CGNs were transfected with AKAR3EV expression vector. Forty eight hours after transfection, 1mM dbcAMP was administrated in the medium and incubated for 15 min. FRET and CFP images were taken every one min. and acceptor photo bleaching was performed by exposing YFP excitation light for one min without using a natural density filter nor diffusion filter. After bleaching, FRET and CFP images were also taken every one min. (B-C) To address CFP bleedthrough into YFP channel, CFP only expressing vector was transfected in CGNs. CFP transfected cells were then excited by 438nm CFP excitation light and fluorescence was recorded in the CFP and YFP emission channel. YFP channel intensity per CFP channel intensity was calculated and found out 0.23 in our system (Coefficient α). To address YFP excitation by CFP excitation light, YFP only expressing vector was transfected in CGNs. YFP transfected cells were then excited by CFP or YFP excitation light and fluorescence was recorded in the YFP emission channel. YFP channel intensity excited by CFP emission per FRET channel intensity excited by YFP emission was calculated and found out 0.01 in our system (Coefficient β). We then calculated corrected FRET values according to the following equation by MetaMorph software. [CorrFRET] = [RawFRET] − β*[Acceptor] − α*[Donor]. (TIF) [file pone.0147466.s002.tif]

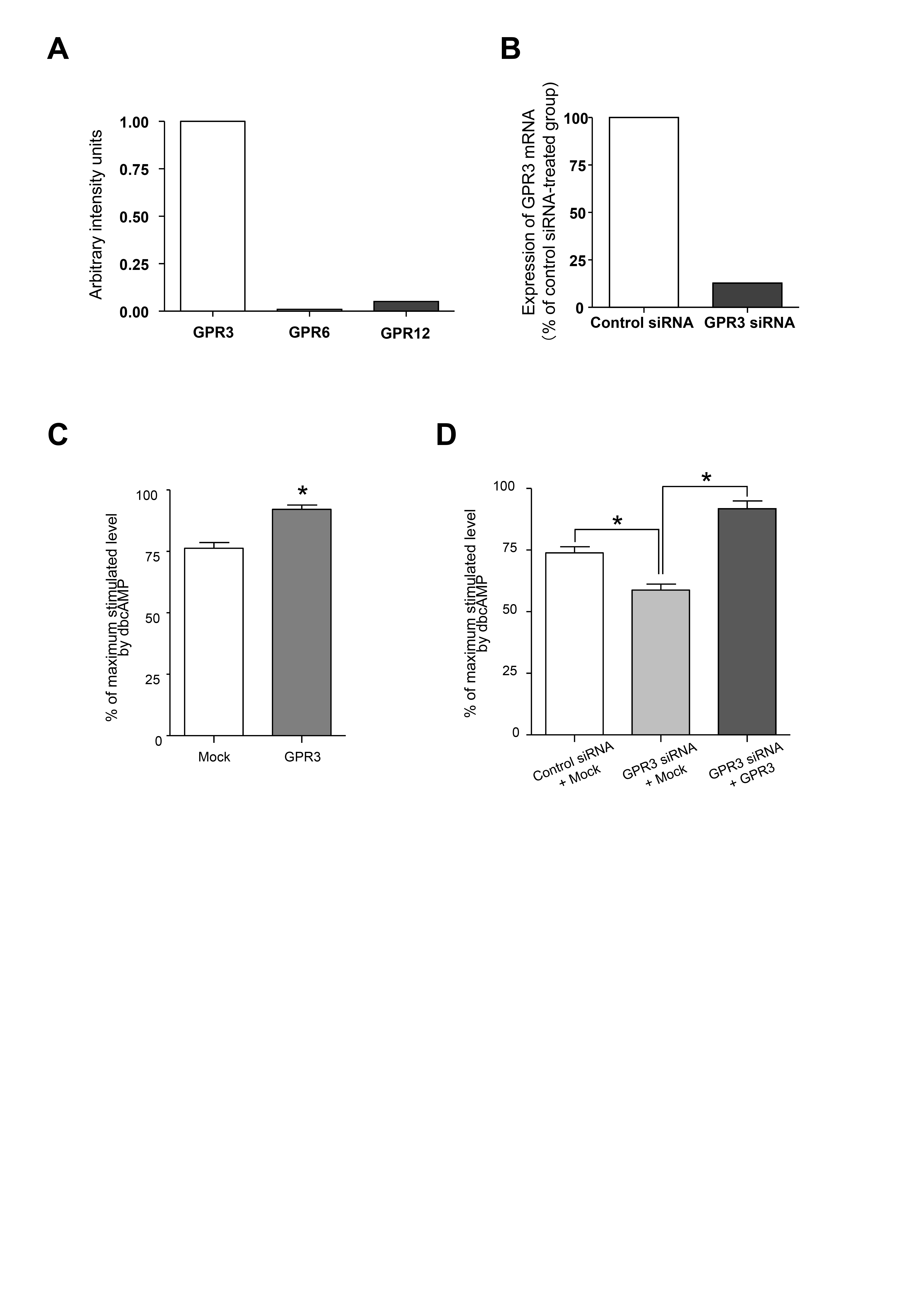

Supplement: S3 Fig — (A) We evaluated the intrinsic expression of GPR3 in SH-SY5Y cells under normal culture conditions. The total RNA was extracted from SH-SY5Y cells in normal culture conditions. The RNA samples were subjected to quantitative RT-PCR analysis using primers specific to human GPR3, GPR6, and GPR12. All quantitative data were adjusted using the levels of the GAPDH mRNA as internal control. Real-time PCR analysis revealed that GPR3 was endogenously expressed in SH-SY5Y cells, but GPR6 and GPR12 were expressed at very low levels in these cells. (B) The SH-SY5Y cells were transfected with a control siRNA or GPR3 siRNA. Twenty-four hours post-transfection, the levels of the GPR3 mRNA was examined by real-time PCR. The endogenous expression of GPR3 was reduced to ~10–15% by the transfection of the GPR3 siRNA. (C) SH-SY5Y cells were co-transfected with AKAR3-EV and a GPR3 expression plasmid. Forty-eight hours after transfection, the FRET/CFP images were captured using a fluorescent microscope. After capturing the images, some cells were treated with 1 mM dbcAMP for 15 min and additional FRET/CFP images were captured to evaluate the fully activated PKA. The FRET/CFP ratio in each group were analyzed identically in each dish, as previously described. The FRET/CFP ratios are expressed as the percentage of the maximum level stimulated by 1 mM dbcAMP in each cell. The data represent the means ± SEM for each condition (n = 8). The asterisk (*) represents p < 0.0001. (D) SH-SY5Y cells were co-transfected with AKAR3-EV and the GPR3 siRNA. Forty-eight hours after transfection, the FRET/CFP images were captured using a fluorescent microscope. For the rescue experiments, a GPR3-expressing plasmid was also co-transfected with the GPR3 siRNA. The FRET value in each condition was evaluated as described above. The data represent the means ± SEM for each condition (n = 6). The asterisk (*) represents p < 0.005. (TIF) [file pone.0147466.s003.tif]
